# Neuronal population representation of human emotional memory

**DOI:** 10.1016/j.celrep.2024.114071

**Published:** 2024-04-08

**Authors:** Dustin Fetterhoff, Manuela Costa, Robin Hellerstedt, Rebecca Johannessen, Lukas Imbach, Johannes Sarnthein, Bryan A. Strange

**Affiliations:** 1 Laboratory for Clinical Neuroscience, Center for Biomedical Technology, Universidad Politécnica de Madrid, IdISSC, Madrid, Spain; 2 Swiss Epilepsy Center, Klinik Lengg, Zurich, Switzerland; 3 Neuroscience Center Zurich, University of Zurich and ETH Zurich, Zurich, Switzerland; 4 Department of Neurosurgery, University Hospital Zurich, University of Zurich, Zurich, Switzerland; 5 Reina Sofia Centre for Alzheimer’s Research, Madrid, Spain; 6 Department of Psychology, University of Zurich, Switzerland

**Keywords:** episodic memory, medial temporal lobe, hippocampus, amygdala, single-units, demixed principal component analysis

## Abstract

Understanding how emotional processing modulates learning and memory is crucial for the treatment of neuropsychiatric disorders characterized by emotional memory dysfunction. We investigate how human medial temporal lobe (MTL) neurons support emotional memory by recording spiking activity from the hippocampus, amygdala, and entorhinal cortex during encoding and recognition sessions of an emotional memory task in patients with pharmaco-resistant epilepsy. Our findings reveal distinct representations for both remembered compared to forgotten and emotional compared to neutral scenes in single units and MTL population spiking activity. Additionally, we demonstrate that a distributed network of human MTL neurons exhibiting mixed selectivity on a single-unit level collectively processes emotion and memory as a network, with a small percentage of neurons responding conjointly to emotion and memory. Analyzing spiking activity enables a detailed understanding of the neurophysiological mechanisms underlying emotional memory and could provide insights into how emotion alters memory during healthy and maladaptive learning.

## Highlights

• Neuronal responses to emotional and neutral stimuli were studied during a memory task
• Amygdala, hippocampal, and entorhinal cortex neurons encoded emotion and memory
• Demixed principal-component analysis separated emotional and memory representations
• Emotional selectivity was detected before memory responsiveness during retrieval

## Graphical abstract

**Figure:**
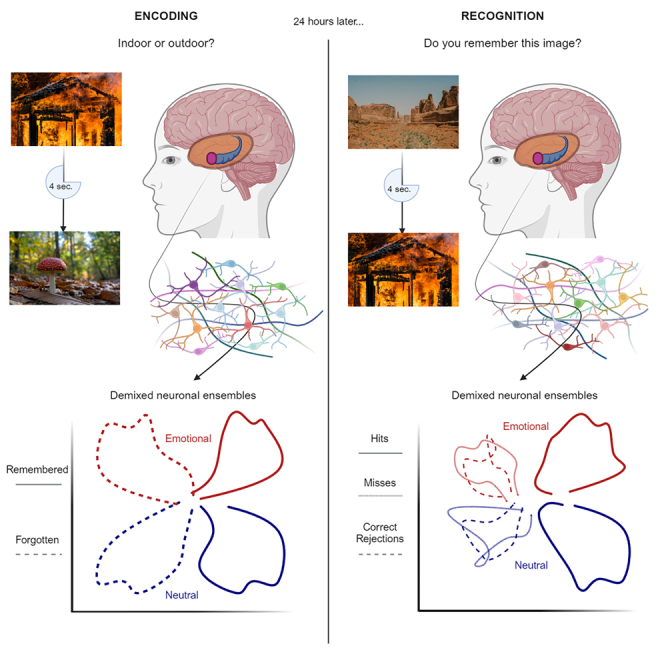

## Teaser

Fetterhoff et al. conducted electrophysiological microwire recordings in the amygdala, hippocampus, and entorhinal cortex of patients with pharmaco-resistant epilepsy during an emotional memory task. They found that neural populations processed both emotional and memory information and used demixed principal-component analysis to segregate emotional and memory representations in the recorded neurons.

## Introduction

Emotional events have a remarkable ability to elicit enduring traces in our memory and form an essential part of our autobiographical history.^1^^,^^2^ Events with strong emotional components, either positive or negative, can often be vividly recalled for one’s entire lifetime. There is substantial evidence that the medial temporal lobe (MTL), including the amygdala and the hippocampus, plays a significant role in emotional episodic memory in both animals^3^ and humans.^1^^,^^2^ In humans, the constraints posed by existing non-invasive neuroimaging methods necessitate the utilization of direct electrophysiological recordings from the amygdala, hippocampus, and surrounding entorhinal cortex to study the neuronal dynamics involved in emotional memory encoding and retrieval. Multiple previous intracranial electroencephalogram (EEG) studies found increased gamma activity to emotional stimuli compared with neutral ones.^4^^,^^5^^,^^6^^,^^7^^,^^8^ While gamma activity might be a proxy for neuronal spiking,^7^ few studies have examined spiking activity related to human emotional memory.

Human hippocampal and amygdala spiking activity has been shown to differentially respond to human facial expressions,^9^ while human amygdala neurons were shown to encode the subjective judgments of emotions conveyed by facial expressions rather than merely the physical attributes of the facial stimuli.^10^ Another recent study showed that human amygdala neuronal spiking is co-modulated with high gamma power and fMRI blood-oxygen-level-dependent (BOLD) activity during videos of fearful faces.^11^ However, less is known about how single cells contribute to emotional scene memory in humans. Therefore, this study was designed to assess the MTL neuronal dynamics involved in emotional scene memory. As with most intracranial human neuronal studies,^12^^,^^13^^,^^14^^,^^15^^,^^16^ we grouped all recorded brain regions together for the main analyses but conducted secondary analyses when segregating by brain region.

We first analyzed single-unit responses before assessing population dynamics using demixed principal-component analysis (dPCA).^17^ We implemented dPCA to succinctly summarize the mixed selectivity in our recorded neuronal population, as dPCA overcomes some shortcomings associated with traditional PCA. While PCA efficiently extracts principal components (PCs) from neural data, it overlooks stimulus- and decision-related information, resulting in mixed selectivity and complex population activity dominated by temporal dynamics. In contrast, dPCA strikes a balance between demixing and compression, effectively separating neural activity related to different task parameters while reducing data dimensionality.

We hypothesized that the recorded neuronal population would effectively distinguish between remembered and forgotten scenes as well as emotional and neutral stimuli. Furthermore, we expected that the hippocampus, recognized for its crucial role in memory functions, would demonstrate responsiveness to memory-related processes, while the amygdala, known for its involvement in emotional processing, would exhibit sensitivity to emotional content. Moreover, beyond the examination of regional differences, our investigation aimed to assess whether the entire MTL neuronal population collectively reflects the intricate interplay between emotional and memory processes. By investigating the neural mechanisms underlying the integration of memory and emotion, we seek to advance our understanding of the fundamental cognitive processes of emotional scene memory encoding and recognition.

## Results

### MTL neurons respond differentially to emotional and remembered stimuli

Findings from research in cognitive science, neuropsychology, and neuroimaging consistently suggest that human memory performance, specifically in recognition memory tasks, can be attributed to two separate memory processes, commonly known as recollection and familiarity.^18^ While familiarity is associated with variable memory strength, recollection involves a threshold retrieval process,^18^ where individuals retrieve “qualitative” information about past events, such as temporal and spatial context and associations between event components. When we focus on the meaning of a stimulus (deep processing; e.g., determining whether a word is concrete or abstract, whether a scene is inside or outside) instead of its perceptual features (shallow processing; e.g., whether it is in upper- or lowercase), it enhances our ability to remember it and, to a lesser extent, our sense of familiarity with it.^18^ We implemented an indoor/outdoor judgment to improve memory performance and examine the neuronal basis of deep emotional memory encoding^18^ by exclusively focusing on remembered (recollected) compared with unremembered events without considering familiarity.

**Figure 1:**
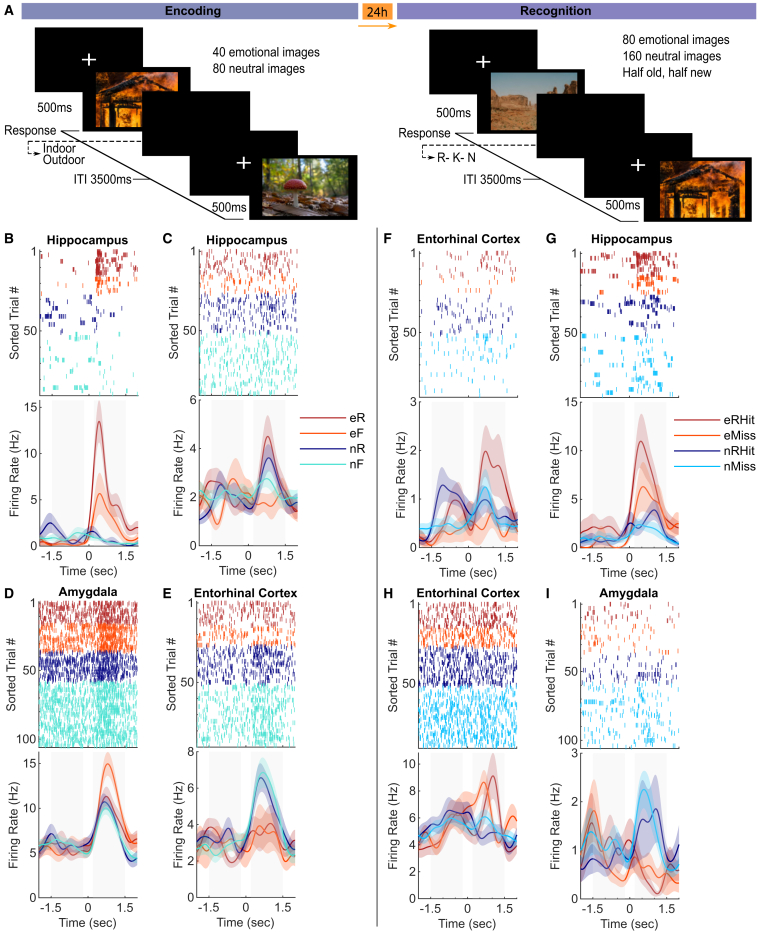
Activity of MTL neurons during emotional memory encoding and retrieval (A) Illustration of emotional memory task with 2 emotion types, emotional (aversive) and neutral, presented during encoding and recognition task phases. Images were taken from Unsplash. (B–G) Trials are sorted by grouping trial types together. Baseline and stimulus time periods are shaded gray. e, emotional; n, neutral; R, subsequently remembered; F, forgotten; RHits, remembered hits; M, misses. CRs were excluded from the example plots because they were similar to misses. Shaded regions are the standard error of the mean (SEM). (B–E) Encoding. (B) Hippocampal neuron with both main effects and an interaction. (C) Hippocampal neuron with a main effect of memory only. (D) Amygdala neuron with a main effect of emotion and trend toward an interaction (*p* = 0.07). (E) EC neuron with a main effect of emotion that responded more strongly to neutral scenes. (F–I) Recognition. (F) EC neuron with a main effect of memory and trend toward an interaction (*p* = 0.06). (G) Hippocampal neuron with both main effects. (H and I) Example neurons with main effects of emotion.

Nine patients (4 males) with drug-resistant epilepsy participated in an incidental emotional memory task (Figure 1A). During encoding, participants responded “indoor” or “outdoor” to 120 images, one-third of which were emotional (aversive). Twenty-four hours later, a period thought sufficient for consolidation of emotional memories,^19^^,^^20^ participants responded Remember (R), Know (K), or New (N) during a recognition memory task that contained all emotional and neutral old images and the same number of new images.^7^ There was a significant main effect of memory showing a significantly greater percentage of remembered vs. false alarm trials for both emotional and neutral trials (repeated-measures ANOVA, F(1,8) = 13.1, *p* = 0.0068), indicating that patients actually remembered the images. There was no interaction between memory and emotion in the analyzed dataset (Table S2) presumably because this study exclusively focused on patients with microelectrodes recording single-unit activity. However, an interaction was detected when including 13 additional subjects with only local field potential (LFP) electrodes.^7^ Due to low numbers of K and false alarm trials (Table S1), these trials were excluded from the primary analyses.

Neuronal responses from 5 patients with acceptable memory performance (Table S2) were analyzed during task performance. Spike sorting^21^ was conducted, and spike quality metrics were assessed (Figure S1). In total, we detected 542 putative single units (referred to as neurons hereafter) in the hippocampus, amygdala, and entorhinal cortex of all patients (258 during encoding and 284 during recognition). During encoding trials, we compared spiking activity between emotionally aversive (e) and neutral (n) scenes, further classified as subsequently remembered (eR and nR) or subsequently forgotten trials (eF and nF); i.e., old items at recognition that received a new response. For recognition trials, we compared firing rate patterns between remembered hits (RHits), misses (Miss), and correct rejections (CR) for both emotional and neutral trials.

To illustrate the diversity of responses during encoding, raster plots and smoothed firing rates of 4 different individual neurons are plotted in Figures 1B–1E. We often observed higher firing rates during encoding for both e vs. n and subsequent R vs. forgotten (F; Figures 1B and 1C), although some neurons exhibited the opposite response type (Figure 1E). The responses of four neurons recorded during recognition illustrate the diversity of neuronal responses, often with some form of emotional differentiation (Figures 1F–1I).

### Summarizing firing rate changes by emotion and memory

**Figure 2:**
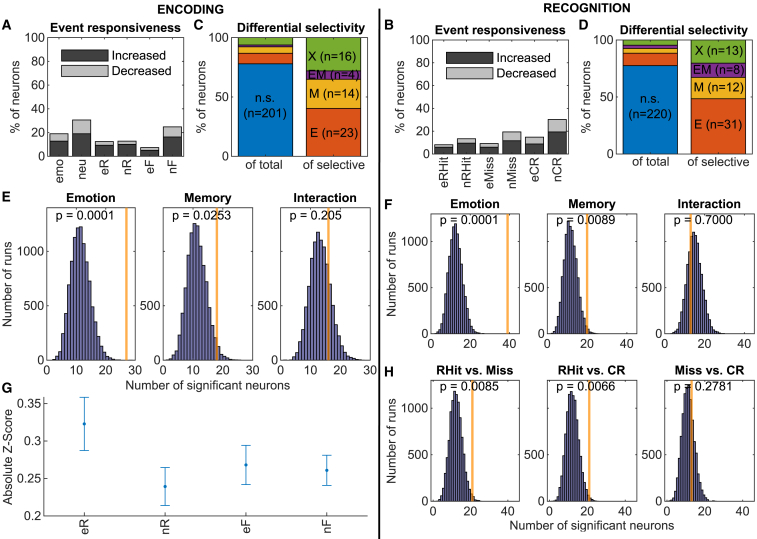
The population of single neurons differentially represented remembered and forgotten images Data are from 5 subjects. (A) Percentage of neurons with significant differences in spike counts between baseline and stimulus during encoding. Trial types with more data have higher chances of showing event responsiveness (i.e., neutral and nF). (B) Same as (A) during recognition. (C) Bar charts showing percentages of selectively responding neurons as a percentage of all neurons (left) and selective neurons (right). n.s., not significant; E, emotional; M, memory; X, interaction. (D) Same as (C) but during recognition. (E) Bootstrapping was used to determine whether the number of observed neurons (orange lines) was above chance. The *p* value is computed as the proportion of chance observations (blue) that surpasses the observed count. See also Figures S2A–S2C and S3. (F) Same as (E) but during recognition. (G) Averaged absolute-valued *Z* scores of all neurons for each trial type used in the linear mixed-effects model. Error bars represent the SEM. (H) Bootstrapping was used to determine whether a significant number of neurons distinguished between pairs of trial types during recognition. See also Figures S2D–S2F.

We first examined stimulus-evoked changes in firing rate (i.e., event responsiveness). We compared raw spike counts between baseline and peri-stimulus time periods (0.2–1.5 s before and after stimulus presentation, respectively) of all conditions using a permutation test. Percentages of neurons with significant firing rate changes are shown for encoding (Figure 2A) and recognition (Figure 2B).

Event responsiveness alone merely indicates whether a neuron responds to a stimulus but provides no insight into differential selectivity when comparing various trial types. We investigated differential responding by analyzing spike counts during the peri-stimulus periods of different trial types. For each neuron, we performed a 2 × 2 repeated-measures ANOVA during encoding (Figure 2C; e vs. n and R vs. F) and a 2 × 3 repeated-measures ANOVA during recognition (Figure 2D; RHits vs. Miss vs. CRs). To determine whether the observed numbers of emotion, memory, or emotional memory processing neurons were above chance, we used a bootstrapping procedure to compare the number of observed cells with a null distribution.^14^ We found significantly more emotion (*n* = 27 encoding, *n* = 39 recognition) and memory (*n* = 18 encoding, *n* = 20 recognition) neurons than expected by chance during both encoding and recognition but not emotional memory (interaction, *n* = 16 encoding, *n* = 13 recognition) neurons (Figures 2E and 2F). This indicates that a significant number of neurons responded differently to remembered compared to forgotten trials during encoding (Figure 2E). During recognition, we found significant differences when comparing RHits to Misses and RHits to CRs, but Misses and CRs were similar (Figure 2H). We performed all pairwise comparisons for know hits (KHits) but did not find any significant differences after correcting for multiple comparisons (Figure S2D). These analyses were repeated for each brain region (Figure S2), by brain region including K trials (Figure S2), and for each subject (Figure S3). During encoding, we found main effects of memory in the hippocampus and entorhinal cortex (EC) and main effects of emotion in the amygdala and hippocampus (Figures S2A–S2C). During recognition, we found main effects of emotion in all 3 brain regions and a main effect of memory in the EC (Figures S2E and S2F).

To quantify variations in firing rates over the pool of recorded neurons, we calculated *Z* scores for each trial type and compared them with two linear mixed-effects models, one for encoding (Figure 2G) and another for recognition, using subject and neuron as nested random intercept effects. During encoding (258 neurons), we found a significant main effect of emotion (χ^2^(1) = 4.31, *p* = 0.038), a significant emotion × memory interaction (χ^2^(1) = 4.05, *p* = 0.044), and a trend toward a main effect of memory (χ^2^(1) = 2.94, *p* = 0.086). Follow-up pairwise comparisons showed that there was a significant increase in firing rates for subsequently remembered compared to subsequently forgotten emotional items (*Z* ratio = 2.339, *p* = 0.019), while there was no such effect for neutral items (*Z* ratio = 0.284, *p* = 0.777). We did not find any significant effects during recognition (data not shown).

### Emotionality interacts with memory during encoding in MTL neuronal populations

**Figure 3:**
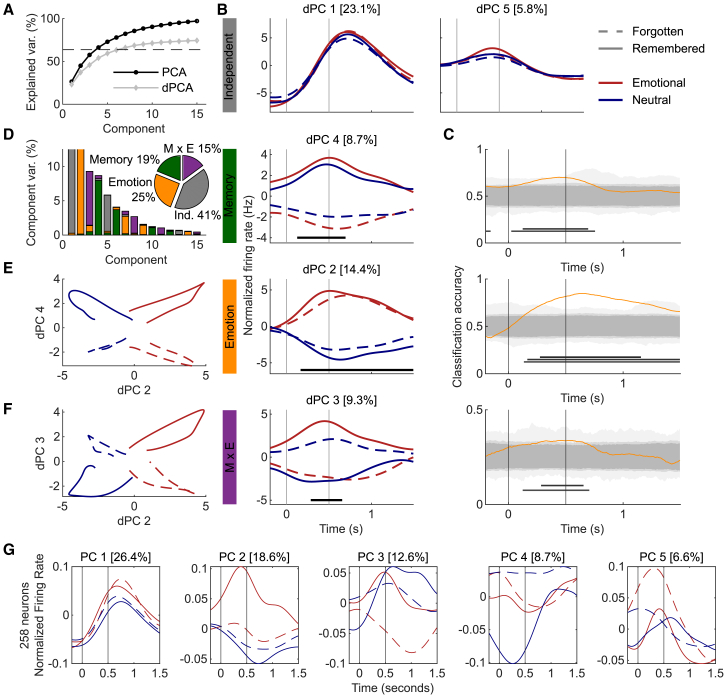
Demixed PCA distinguished all trial types during emotional memory encoding Data are from 5 subjects and 258 neurons. (A) The cumulative explained variance (var.) of the first 15 dPCA components surpassed the estimated signal variance (dashed line), and the largest 4–6 dPCs explain similar variance to their PCA counterparts. (B) dPCs are plotted as a function of peri-stimulus time. Vertical lines represent stimulus onset (0 s) and offset (0.5 s). Horizontal black bars represent time periods when the real classification accuracy surpassed 97.5% of the shuffled accuracies. Top row: condition-independent components represent changes in neuronal firing rate time-locked to stimulus presentation. Second row: differences in memory, subsequently remembered vs. forgotten, began during stimulus presentation. Third row: differences between emotion (emotional vs. neutral) began during stimulus presentation. Bottom row: an interaction between memory and emotion was detected in a short time window near stimulus offset. (C) Cross-validated time-dependent classification accuracy of linear classifiers (orange) was compared with accuracy from 1,000 shuffled distributions (gray regions). Time windows when real accuracy surpassed 100%, 97.5%, and 95% (*p* < 0.05, two-tailed) are defined (black lines) below the respective distributions. (D) Component variance (%) is the percentage of total variance explained by the first 15 components. The first 5 bars are each predominantly one color, indicating an acceptable level of component demixing. The vertical axis is truncated to show the distribution of variance, and the highest variances are in (B) titles. Pie chart percentages are normalized to the explained variance of the first 15 components. Ind., independent. (E and F) Plotting the normalized firing rate of 2 dPCs with significant time windows revealed separation of the 4 trial types. (G) Regular principal components (PCs) explained more variance than dPCs but did not effectively separate trial types.

**Figure 4:**
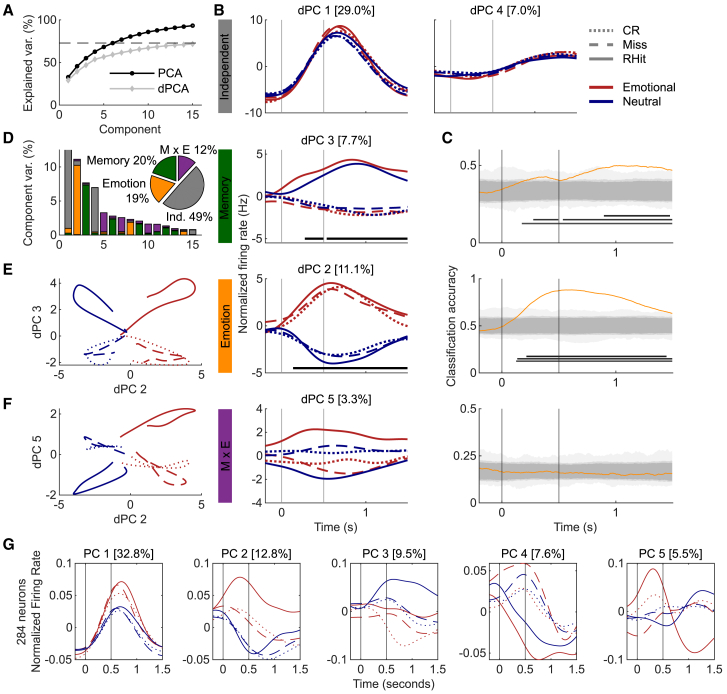
Demixed PCA distinguished memory response and emotion during emotional memory recognition Data are from 5 subjects and 284 neurons. The plot layout matches Figure 3 but with recognition neurons. (A) Same as Figure 3A. (B and C) The emotional effect begins before the memory effect. (D) Same as Figure 3D. (E and F) Plotting the normalized firing rate of 2 dPCs revealed sepration of RHits from Misses and CRs, which were consistently overlapping. (G) Regular principal components (PCs) explained more variance than dPCs but did not effectively separate trial types.

We characterized the mixed selectivity of the MTL neuron population by projecting the firing rate across the entire population of MTL neurons on demixed PCs (dPCs) by dPCA.^21^ A key advantage of dPCA lies in its ability to incorporate all neuronal data without imposing significance thresholds on individual neurons (that could eliminate borderline neurons like in Figures 1D and 1F) to determine how an entire population represents behavioral variables. We used dPCA to decompose neuronal population activity into components concerning task parameters (stimulus onset and offset), memory performance, and emotion (emotional vs. neutral) during encoding (Figure 3) and recognition (Figure 4).

During encoding, the cumulative explained variance of the first 15 dPCs exceeded the estimated signal variance (Figure 3A), indicating that the unexplained variance is likely due to noise.^17^ The first four dPCs explained 55.5% of the variance and demonstrated several important findings (Figure 3B). The largest component, dPC1, explained over 23% of the variance associated with condition-independent sources, reflecting neurons’ altering firing rates across all trial types, possibly indicative of event responsiveness (Figures 2A and 2B). Overall, condition-independent components, including dPC 1, accounted for 41% of the normalized explained variance (Figure 3D). Emotion emerged as the second largest variable, constituting 25% of the normalized explained variance (Figure 3D). Notably, dPC 2 clearly distinguished emotional from neutral stimuli during the stimulus presentation, constituting over 14% of the explained variance (Figure 3B). dPC 3 captured the interaction between emotion and memory during the stimulus presentation (Figure 3B). dPC 4 highlighted memory-related activity during the stimulus presentation (Figure 3B). Significant time windows were defined when the real classification accuracy surpassed 97.5% of 1,000 shuffled accuracies (Figure 3B) and at several significance thresholds (Figure 3C).

A visual representation of the largest emotion (dPC 2) and memory (dPC 4) components plotted against each other revealed a clear separation between the four trial types in component space (Figure 3E). Similarly, plotting the interaction component (dPC 3) against the emotional one (dPC 2) illustrates separation of all trial types (Figure 3F). Collectively, these three dPCs revealed differential firing rate components for all four combinations of emotion and memory during encoding and roughly corresponded to the percentages of neurons with similar response characteristics (Figure 2C).

Additionally, since PCA explained more variance than dPCA, we plotted the first 5 regular PCs to illustrate the differences between the two methods (Figure 3G). PC 1 resembled dPC 1 but with some emotional effect, illustrating proper demixing in dPC 1. PC 2 illustrates the strong eR responding, while PCs 3–5 are less readily interpretable. Finally, we performed a leave-one-out analysis to examine the contribution of each recorded brain region but did not find strong evidence of any region performing a single function (Figure S4, top).

### Emotion and memory are distinguishable in MTL neuronal population activity during recognition

We next extracted dPCs from the neuronal activity during recognition, which revealed different yet complementary aspects of the neuronal population activity (Figure 4). First, the overall explained variance of the first 15 dPCs reached a comparable level as in encoding (compare Figures 3A and 4A). The largest dPC was again related to condition-independent activity (Figure 4B, top row), with dPC 1 peaking just after stimulus offset. Additionally, dPC 4 peaked near the average reaction time of all subjects (1.65 s, std = 0.54 s). Accordingly, condition-independent components captured nearly half of the normalized component variance (Figure 4D). dPC 2 captured the different responses to emotional compared with neutral images beginning early during stimulus presentation and extending until at least 1.5 s after stimulus presentation (Figure 4B). Population activity captured by dPC 3 reflected a difference between images that were remembered (RHit) compared with both misses (old images judged as new) and CRs. No significant interaction between emotion and memory was detected during recognition (Figure 4B). The decoding accuracy increased for emotion earlier than for memory, indicating that emotional differences were detected before memory ones (Figure 4C). Plotting the task-related dPCs against each other revealed clear differences between RHits compared to both misses and CRs, with the latter two consistently overlapping in component space (Figures 4E and 4F). This suggests that MTL neurons represent subjective memory over objectively distinguishing old from new images, consistent with a previous report.^14^

We again plotted the first 5 regular PCs to illustrate the differences between the two methods (Figure 4G), and the overall trend was similar to encoding with PC 1 aligning with dPC 1 except mixing condition-independent activity with emotional responding. As during encoding, PC 2 differentiated emotional RHits from all other trial types. Finally, we performed a leave-one-out analysis to examine the contribution of each recorded brain region and found further evidence that the hippocampus and EC contributed most strongly to the memory effects (Figure S4, bottom), consistent with the previously mentioned memory effects (Figures S2D and S2E). We also found the biggest deterioration of emotional selectivity by removing the hippocampus (Figure S4, bottom).

## Discussion

Our findings emphasize that neural populations within the human MTL are actively engaged in concurrent memory and emotional processing during encoding and retrieval, largely independent of specific MTL regions. Consistent with our brain region hypotheses, we observed emotion-related neuronal activity in all recorded MTL regions and memory-related activity in both the hippocampus and EC (Figure S2). Our results align with a growing body of human intracranial research, which consistently integrates multiple MTL regions^12^^,^^13^^,^^14^^,^^15^^,^^16^ to unveil novel insights into mnemonic processing. This collective evidence strengthens the well-established role of the MTL in emotional memory^7^^,^^10^^,^^22^^,^^23^^,^^24^ and underscores the functional significance of the EC.^25^^,^^26^^,^^27^ Our results support the hypothesis that the human MTL responds to its perceived environment^9^ to relate and segregate experiences using mixed selectivity of neuronal responses that are subsequently read out by downstream brain regions to enable conscious experience.

To illustrate the various forms of mixed firing rate selectivity in the recorded population, firing rate patterns of all recorded neurons were qualitatively depicted (Figures 1 and 2) before quantifying the population firing rate dynamics using dPCA (Figures 3 and 4). dPCA allows the heterogeneity of neuronal responses to be incorporated into one unified description of task-related population activity.^17^ dPCA separated memory and emotional representations to provide further evidence that MTL neuronal populations process both types of information. One noticeable difference between the dPCA results for encoding and recognition is that the condition-independent components, which are components representing changes in firing rates irrespective of memory or stimulus type and therefore related to event responsiveness (Figures 2A and 2B), explained more variance during the recognition test (Figures 3B, 3C, 4B, and 4C, gray). During recognition, participants are actively performing memory retrieval before they press a button to make their responses. Interestingly, the second normalized firing rate peak in dPC 4 occurred prior to the average reaction time of about 1,650 ms (Figure 4B), but no such component appeared during encoding without an explicit memory task and when reaction times were shorter and less variable.

Differences in the latency to respond to emotion and memory in the recorded neuronal populations were also revealed by dPCA. Namely, differences between emotional and neutral stimuli were detected earlier than differences between remembered and forgotten stimuli during retrieval (Figure 4). This finding corresponds to scalp EEG results showing that event-related potential (ERP) differences occur earlier between emotional and neutral hits than an old/new (memory) effect.^28^ Emotional features must be perceived quickly to modulate attention or behavior,^29^ and the fact that emotional salience is perceived before memory judgments is consistent with the sequence of events required for emotional enhancement of memory.^30^ Alternatively, during encoding, these effects occurred nearly simultaneously with the significant interaction component (Figure 3B).

We found that, on average, neurons exhibited higher firing rates during the encoding of subsequently remembered items (eR/nR) compared with subsequently forgotten ones (eF/nF). This finding is consistent with the difference due to memory hypothesis^31^ and may reflect successful memory encoding. Additionally, the significant interaction between emotion and memory detected during encoding using linear mixed-effects models and dPCA could be related to the frequently observed memory enhancement for emotional compared to neutral items.^7^^,^^23^ Although we did not find a memory enhancement for emotional images in our patient sample (Table S2), the dPCA results suggest that there is a neuronal mechanism for this enhancement in MTL neurons near stimulus offset (Figure 3), a time window consistent with the previously detected emotion by memory interaction in amygdala gamma activity.^7^ Interestingly, the timing of the encoding interaction is similar to the late positive potential (LPP) ERP effect in scalp EEG that typically begins around 300–400 ms post stimulus presentation.^32^ Besides timing, the LPP shares other characteristics with our effects as it is larger for emotional than neutral stimuli^33^ and is enhanced for subsequently being remembered compared to subsequently forgotten stimuli during encoding.^34^

We found a significant interaction between emotion and memory during encoding using the linear mixed-effects model and dPCA, but only a trend in the behavioral results and not when counting the number of neurons. Since emotional scenes were better remembered than neutral scenes in our previous study with a large sample,^7^ the present results emphasize the importance of using population-based analyses to understand neuronal network dynamics over selecting neurons based on individual firing rates for further analysis.^17^ We did not find a significant number of neurons exhibiting an emotional memory interaction, which is consistent with the idea of “concept cells,” where neurons with unique, discrete representations form associations or networks to represent larger concepts.^35^

Contrary to encoding, no significant interaction between emotion and memory was detected using dPCA during recognition despite the E × M interaction representing 12% of the explained variance (Figure 4D). However, the recognition dPCA results suggest that misses (old items judged as new) and CRs (correctly identified new items) are similarly represented in the recorded neuronal population (Figures 4B–4E, dPC 3, for example). This suggests that MTL neurons represent a subjective component of memory experience (the feeling that something is new) instead of a veridical old/new distinction, which is consistent with previous neuronal and fMRI findings.^14^^,^^36^

### Limitations of the study

A major limitation of this study is the small sample size, which prevented us from drawing stronger conclusions about differential brain region computations. We cannot exclude the possibility that the absence of a significant number of neurons showing an emotional memory interaction during encoding is related to our relatively small sample size. Additionally, human single-neuron recordings are only performed in people with intractable epilepsy who can have poor overall memory performance.^37^ Although statistically above chance level, the performance in this cohort was relatively low (Table S2) and lower than that of a larger cohort of patients undergoing intracranial recordings performing the same task.^7^ Future experiments could attempt to improve memory performance by increasing the presentation time or limiting recognition judgments to old/new. Another limitation is that, since encoding and retrieval were performed 24 h apart, it is highly unlikely that we recorded the same neurons at both phases. Since emotional modulation of memory consolidation is thought to require sleep,^19^ it is difficult to shorten the encoding-retrieval delay to perform representational similarity analysis on neuronal populations, as described recently.^38^

### Conclusions

Previous studies have shown that remembering emotionally aversive events requires collaborative activity between cortical and subcortical MTL regions that enable mnemonic processing.^1^^,^^7^^,^^23^^,^^27^ Although a nonsignificant number of neurons demonstrated selective firing for the conjoint features of emotion and memory performance, we found a significant emotional memory interaction during encoding in the firing rate components extracted from population activity of neurons in the amygdala, hippocampus, and EC. Understanding human neuronal network dynamics can ultimately yield novel clinical insights.^39^ Improving our understanding of the neurophysiological mechanisms underlying emotional memory can provide insights not only into the pathogenesis of psychiatric disorders characterized by maladaptive enhancement of emotional components of memory,^40^ as in post-traumatic stress disorder (PTSD)^41^ and phobia, but also for epilepsy patients, where memory networks are disrupted by resective surgery^42^^,^^43^ and deep brain stimulation.^44^^,^^45^

## STAR★Methods

### Key resources table

**Table:** 

REAGENT or RESOURCE	SOURCE	IDENTIFIER
**Deposited data**		

Spike trains & waveforms	This paper	https://doi.org/10.12751/g-node.q66jki

**Software and algorithms**		

MATLAB	MATLAB 2022b	http://www.mathworks.com
Combinato spike sorting	Performed in Python 3.8	https://github.com/jniediek/combinato
FieldTrip toolbox	Performed in MATLAB 2022b	https://doi.org/10.1155/2011/156869
Demixed PCA	Performed in MATLAB 2022b	https://github.com/machenslab/dPCA
Custom MATLAB code	This paper	https://doi.org/10.5281/zenodo.10782334
lme4 package	Performed in R (v4.2.2) with RStudio (v2022.12.0)	https://github.com/lme4/lme4

**Other**		

Macro/micro depth electrodes	Ad-Tech	www.adtechmedical.com
ATLAS recording system	NeuraLynx	ATLAS

### Resource availability

#### Lead contact

Further information and requests for resources and reagents should be directed to and will be fulfilled by lead contact Dustin Fetterhoff (dustin.fetterhoff@ctb.upm.es).

#### Materials availability

This study did not generate new unique reagents.

#### Data and code availability

• All spike trains and waveforms collected during the experiments have been deposited on GIN in MATLAB format (https://gin.g-node.org/dfetterhoff/emotional_memory_neuronal_data to be used with the FieldTrip toolbox) that can be run to reproduce all result and figures using our scripts on GitHub (https://github.com/dustinf1989/emotional_memory_neuronal_analyses). DOIs are listed in the key resources table. Ethical approval and informed consent obtained from patients did not include the publication of raw patient data, thus raw signals from intracranial microwires cannot be shared.
• Any additional information required to reanalyze the data reported in this paper is available from the lead contact upon request.

### Experimental model and subject details

Participants were patients with medication-resistant epilepsy undergoing pre-operative diagnostic monitoring with implanted electrodes in the medial temporal lobe (9 participants, aged 25–55, 4 male). All participants had normal or corrected-to-normal vision, were not color-blind, and were right-handed as validated by neuropsychological testing. All patients had borderline to normal IQs ranging from 71 to 109, and there was no correlation between IQ or neuropsychological tests and task performance. All patients signed informed consent and did not receive financial compensation. All participation was voluntary, and participants had the right to withdraw their consent at any time during the experiment. The study followed the declaration of Helsinki for medical research involving human subjects and had full approval from both the local ethics committee of Kantonale Ethikkommission, Zurich, Switzerland (PB-2016-02055) and the European Research Council Ethics Board.

### Method details

#### Surgical procedure

Depth electrodes (1.3 mm diameter, 8 macro-contacts of 1.6 mm length and spacing between contact centers 5 mm; Ad-Tech, Racine, WI, www.adtechmedical.com) were stereotactically implanted into the hippocampus, amygdala, and entorhinal cortex. Nine microelectrodes extended approximately 4 mm from the tip of the depth electrode. We use the term "Entorhinal Cortex" to describe the neuroanatomical target of medial temporal cortical electrodes, and note that, due to inter-patient variability in anatomy, particularly of the collateral sulcus, some wires may be in perirhinal cortex.

#### Electrode contact localization and visualization

Electrode localization was performed for each patient using the following pipeline: 1) co-registration of pre-electrode placement T1-weighted magnetic resonance images (pre-MRI) with the post-electrode placement CTs (post-CT). 2) CT and MRI images were skull-stripped, respectively by i) filtering out all voxels with signal intensities between 100 and 1300 HU and by ii) spatially normalizing the image to MNI space employing the New Segment algorithm in SPM8 (http://www.fil.ion.ucl.ac.uk/spm). 3) The resultant inverse normalization parameters were then implemented to the brain mask supplied in SPM8 and the mask was converted into the native space. 4) We filtered out all voxels in pre-MRI lying outside the brain mask and with a signal value in the highest 15th percentile. 5) The skull-stripped pre-MRI was then co-registered and re-sliced to the skull-stripped post-CT. 6) The pre-MRI was normalized to the post-CT, thus transforming the pre-MRI image into native post-CT space. 7) Finally, we thresholded the post-CT to only visualize electrode contacts and overlaid the two images. Contact visualization was performed using Lead-DBS,^46^ To reconstruct and visualize the electrodes, we first selected the electrode model (Ad-Tech), co-registered CT to MRI using the advanced normalization tools (ANTs) and volumes were normalized into MNI ICBM 2009b nonlinear asymmetrical space based on preoperative MRI.^47^ The software also corrects for brain shift. We pre-reconstruct electrodes using the manual reconstruction. This involved marking the tip of each electrode and another point along the electrode trajectory manually. Following this, an automatic reconstruction was executed based on the electrode model (number of contacts and their spacing was considered). The reconstructed electrodes underwent visual inspection, and, in case of any misalignments, we manually refined the reconstruction based on postoperative CT by placing the trajectory as precisely as possible within the center of the electrode artifact.^46^ Using Lead-group we visualized all electrodes and saved the MNI coordinates for each contact. Finally, we localized the microelectrode wires by projecting the three-dimensional extension from the electrode tip (Figure S1).

#### Stimuli

Behavioral, local field potential, and anterior hippocampal neurons were published as “Cohort 2” in a previous manuscript^7^ where methods are described in more detail and briefly summarized here. Participants were shown 40 emotional and 80 neutral color scenes during the encoding session. One day later, all old images were intermixed with the same number of new emotional and neutral images during the recognition session to yield 120 old and 120 new images. All 80 emotional images were highly arousing and aversive (mutilations, attacks, guns, blood, etc.) scenes selected from the International Affective Picture System (IAPS).^48^ 149 neutral images were taken from IAPS (neutral people and household scenes) and 11 neutral landscape pictures were obtained from the internet. Mean normative IAPS picture ratings (s.e.m.) on a 9-point scale for valence were 5.05 (±0.05) for neutral, and 2.04 (±0.05) for emotional pictures (lower ratings are more negative), and for arousal were 3.29 (±0.06), and 6.3 (±0.07) for neutral and emotional pictures (higher ratings are more arousing), respectively.

#### Behavioral task

Prior to signing informed consent, patients were shown one emotional (aversive) IAPS picture and notified that they would see similar images on both that and the subsequent day. Task instructions were provided verbally and on-screen in German. Encoding and recognition sessions were performed during the second and third postoperative days (Figure 1A). Memory encoding was incidental, as participants were not informed about a memory test until immediately before the recognition session. During both encoding and recognition sessions, emotional and neutral images were pseudo-randomly presented (presentation time 0.5 s; interstimulus interval 3.5 s) with a constraint that at least one neutral image was presented between emotional ones. Images were presented on a laptop about 50 cm from the subject. During encoding, participants made an indoor-outdoor judgment for each picture using the laptop keyboard with labeled keys. During recognition, participants made a “remember,” “know,” or “new” decision (R-K-N).^7^ “Remember” decisions were made if they could recall the exact image, while “know” decisions were made if an image seemed familiar. During both recording sessions, participants were as still as possible while looking at the fixation cross in the center of the screen and avoiding verbalizations.

#### Data acquisition

Intracranial EEG data were recorded against a common intracranial reference with the ATLAS system (0.5- to 5000-Hz passband, Neuralynx, Bozeman, MT, USA; www.neuralynx.com) with a sampling rate of 30,000 Hz (Patients 1 and 2) or 32,768 Hz (Patients 5, 6, 8, 10, 11, 12 and 13) for each microelectrode and 4,000 Hz or 4,096 Hz for macroelectrodes.

### Quantification and statistical analysis

#### Spike sorting

Spike sorting was performed using the default settings of the Python package Combinato^21^ for each encoding and recognition session independently to identify putative single units termed neurons throughout this report. No attempt was made to merge neurons between encoding and recognition sessions since they occurred 24 h apart, an interval too long to reliably claim to have the same neuron using microwires. Post-sorting, autocorrelograms, and interspike interval (ISI) histograms were inspected to manually eliminate artifactual clusters. Units with noisy waveforms and nonuniform shapes were removed. Only units with less than 3% of ISIs under 3ms, a mean firing rate >0.25 Hz, and at least one spike on 50 or 100 trials during encoding or recognition trials, respectively, were included in subsequent analyses. Artifact spikes due to mechanical or electrical noise occurring 3 ms before or after stimulus appearance or disappearance were removed. Spike sorting quality was assessed with the percentage of interspike intervals less than 3ms (Figure S1). The spike width, equal to peak – trough in ms, was computed to assess the distribution of waveform shape. Due to problems with the recording equipment, no units were detected in Patient 11 and therefore we only included this patient’s behavioral data.

#### Single unit analyses

We excluded 3 more patients from all neuronal analyses due to abnormal or poor behavioral performance (Tables S1 and S2). Patient 2 made all responses around 500 ms, coinciding with stimulus offset, and only chose Know once. Patients 10 and 12 were excluded for poor memory performance because they had PR and d’ values near zero. All behavioral differences were similar in the reduced sample (Table S2). All neuronal analyses were performed in 5 patients.

Event responsiveness was assessed by comparing spike counts between baseline (1.5–0.2 s before stimulus onset) and post-stimulus time (0.2–1.5 s after stimulus onset) windows (gray shaded areas in Figures 1B–1I) using a permutation test by shuffling labels 1,000 times. Event responsiveness was computed for all trial types in Figure 2A for encoding: emotional, neutral, emotional subsequently remembered (eR), neutral subsequently remembered (nR), emotional subsequently forgotten (eF), and neutral subsequently forgotten (nF); and in Figure 2B for recognition: eRHits, nRHits, emotional missed (eM), neutral missed (nM), emotional correct rejections (eCR), and neutral correct rejections (nCR). Percentages of neurons responding were determined using a p < 0.0.5 threshold to illustrate the stimulus responding in the recorded population (Figures 2A and 2B).

Differential selectivity was examined by comparing spike counts using 2x2 (during encoding) and 2x3 (during recognition) repeated measures ANOVA (Figures 2C and 2D) for each neuron. We discarded all main effects when a significant interaction was detected for any neuron. A bootstrapping procedure was used to determine whether the number of observed neurons was significantly above chance.^14^ We calculated the null distribution by performing the same selection process 10,000 times after shuffling the labels associated with each trial in a random manner to compare the bootstrapped data to the actual count of significant cells. This shuffling eliminated any connection between the spiking response and the identity of the trials while maintaining the number of each trial type. The p value was calculated by determining how many chance observations (blue histograms) surpass the observed count (orange lines in Figures 2E, 2F and 2H). After finding a significant main effect of memory during recognition, we performed all 6 possible pairwise analyses using an identical procedure and corrected for multiple comparisons using a Bonferroni correction (p < 0.0086) in Figures 2E and S2D. In instances where no chance values exceeded the observed count, we assigned p values as 1 divided by the number of bootstrap runs (i.e., p = 1/10000 = 0.0001). This procedure was repeated after segregating the dataset by brain region (Figure S2) and by subject (Figure S3).

#### PSTHs and linear mixed effects model

To generate peri-stimulus time histograms, spike trains were filtered with a Gaussian kernel (σ=50 ms) and binned every 10 ms creating single-trial firing rate vectors that were averaged in each condition to produce smooth peri-stimulus time histograms (PSTHs; Figures 1B–1I, bottom panels). These PSTHs were entered into both a linear mixed effects model and demixed PCA. To quantify overall neuronal activity using a mixed effects linear model, z-scores were computed for each trial:

$$z=\frac{x-\;\mu }{\sigma }$$

With x representing the mean of the stimulus period over all trials for the respective stimulus type and both μ and σ derived from the mean and standard deviation of the baseline period from all trials. If the average z-score over all trials was negative, the trial-by-trial z-scores were multiplied by −1 to account for neurons that decreased their firing rates (abs(z)). The trial-by-trial z-scores were used as inputs to a mixed-effects linear model (Figure 2G).

We analyzed the effect of emotion and memory on this absolute z-scored firing rate measure using linear mixed-effects models as implemented in the lme4 package^49^ in R. The model included Emotion (emotional vs. neutral) and Memory (encoding: remembered vs. forgotten; recognition: remembered vs. miss vs. correct rejection) as fixed effects and a nested random intercept effect of PatientID and neuron. We used the “(1|Patient/Neuron)” random effect structure in this study to account for the fact that neurons are nested within patients in our data. Encoding and recognition data were analyzed separately. We tested the significance of the fixed effects and their interaction with omnibus χ^2^ Wald test as implemented in the car package.^50^ The model formula was: abs(z) ∼ 1 + Emotion ^∗^ Memory + (1|PatientID/Neuron).

Pairwise comparisons were corrected for multiple comparisons using the Benjamini & Hochberg false discovery rate (fdr) method.^51^ Additionally, the model was computed using spike times without a Gaussian filter and yielded the same statistically significant differences.

#### Demixed principal component analysis

Demixed principal component analysis (dPCA) deconstructs neuronal ensemble activity into labeled components forming a concise and transparent overview of the population representation.^17^ dPCA overcomes the major shortcomings of PCA.^17^ While PCA efficiently extracts principal components (PCs) from neural data, it does so without considering stimulus and decision-related information, resulting in mixed selectivity. This limitation creates a complex representation of population activity dominated by temporal dynamics. In contrast, dPCA strikes a balance between two crucial objectives: demixing and compression. Demixing involves separating neural activity related to different task parameters, while compression aims to reduce data dimensionality while preserving original information. When comparing linear discriminant analysis (LDA), PCA, and dPCA, it becomes evident that LDA is adept at demixing but distorts the data’s geometry, while PCA excels at compression but fails to separate stimuli effectively.

dPCA introduces an innovative approach by assuming a separate encoder axis for data reconstruction. This flexibility allows dPCA to simultaneously achieve demixing and compression objectives. It selects a decoder axis that reconciles these goals, effectively separating stimuli and preserving the data’s geometrical arrangement. The resulting projection maintains fidelity to the original data, overcoming the trade-off between demixing and compression seen in PCA and LDA. Ultimately, dPCA provides a solution that addresses both the complexity of neural representations and the mixing of stimulus and decision-related information.

The full analysis and software toolboxes for MATLAB and Python are described in detail in the original publication^17^ and summarized here where we used the default algorithm parameters. Smoothed PSTHs (see Linear Mixed Effects Model) were used as inputs to the dPCA algorithm to project neuronal firing patterns aligned to task-related activity onto a low-dimensional component space to summarize population activity. The MATLAB toolbox was adapted to examine effects of emotion (E: emotional vs. neutral), memory (M: remembered vs. forgetting in encoding; remembered vs. missed vs. correct rejections in recognition), and their interaction (E x M). Condition-independent components reflect population-wide firing rate modulations occurring due to temporal characteristics of the task, such as stimuli presentation and physical responses (Figures 3B and 4B, top rows). The “signal variance” (dashed line in Figures 3A and 4A) estimates the level of potentially explainable variance, whereas the remaining variance can be considered noise in the data.

To determine if the differences between individual dPCs were statistically significant, we classified conditions using each dPC as a linear decoder. Horizontal black lines below component-time vectors signify time windows where the respective task parameters were reliably separated from the neuronal population (Figures 3B, 3C, 4B and 4C). Significant time windows were computed using 1,000 iterations of stratified Monte Carlo leave-group-out cross-validation by shuffling trial type labels between conditions 1,000 times using a stratified procedure to match the number of trials per condition (see “Cross-validation to measure classification accuracy” in^17^). Significant time windows were defined where the real classification accuracy surpassed 97.5% of shuffled decoding accuracies (Figures 3B and 4B). The 100%, 97.5%, and 95% (p < 0.05, two-tailed) areas of the shuffled distributions are also illustrated with gray shading from lighter to darker (Figures 3C and 4C). Regular PCA was performed using a singular value decomposition method included in the dPCA toolbox. The leave-one-out analyses were performed the same way except after excluding all neurons from the specified brain region (Figure S4).

## Supplemental information

Document S1. Figures S1‒S4 and Tables S1 and S2

Document S2. Article plus supplemental information
