# Supplementary material for: Neuronal population representation of human emotional memory
Source: Cell Rep. 2024 Apr 8;43(4):114071. doi: 10.1016/j.celrep.2024.114071 (PMC11063625; doi:10.1016/j.celrep.2024.114071)
Supplement: Document S1. Figures S1‒S4 and Tables S1 and S2 [file mmc1.pdf]

Cell Reports, Volume *43*

Supplemental information

Neuronal population representation of human emotional memory

Dustin Fetterhoff, Manuela Costa, Robin Hellerstedt, Rebecca Johannessen, Lukas Imbach, Johannes Sarnthein, and Bryan A. Strange

Subject	eRHit	eRFA	eKHit	eKFA	eMiss	eCR	nRHit	nRFA	nKHit	nKFA	nMiss	nCR
Patient1	21	15	11	17	6	5	4	3	21	15	51	62
Patient2	4	2	0	0	36	38	9	4	1	0	69	75
Patient5	15	7	13	13	12	19	24	6	18	16	38	58
Patient6	12	1	5	8	23	31	33	7	16	14	30	59
Patient8	4	4	18	21	18	15	12	3	23	11	40	64
Patient10	9	10	1	1	26	27	3	2	2	1	72	71
Patient11	11	2	14	17	14	21	23	10	14	15	43	55
Patient12	30	26	3	7	7	7	9	12	18	23	53	45
Patient13	16	7	4	9	20	24	22	8	9	12	47	59

Table S1. Number of trials per response type, related to STAR Methods. Two subjects responded "familiar" (K: Know) very few times (gray cells). Additionally, the number of both emotional (e) and neutral (n) "remember" false alarms (eRKA and nRKA) was only larger than 2 in 4/9 patients (yellow cells). For these reasons, we focus on comparing neuronal responses for correctly remembered images (RHits) to misses (eM & nM) and correct rejections (eCR & nCR). During encoding, we found that subjects were engaged in the task because they responded with indoor/outdoor judgements to 97.3% of all trials (data not shown).

Sub	Hits	FA	PR all	d' R all	eRHit	eRFA	PR eR	d' eR	nRHit	nRFA	PR nR	d' nR
1	20.8%	15.0%	5.8%	0.2242	52.5%	37.5%	15.0%	0.3813	5.0%	3.8%	1.3%	0.1356
2	10.8%	5.0%	5.8%	0.4094	10.0%	5.0%	5.0%	0.3633	11.3%	5.0%	6.3%	0.4315
5	32.5%	10.8%	21.7%	0.7817	37.5%	17.5%	20.0%	0.6159	30.0%	7.5%	22.5%	0.9151
6	37.5%	6.7%	30.8%	1.1824	30.0%	2.5%	27.5%	1.4356	41.3%	8.8%	32.5%	1.1352
8	13.3%	5.8%	7.5%	0.4581	10.0%	10.0%	0.0%	0	15.0%	3.8%	11.3%	0.744
10	10.0%	10.0%	0.0%	0	22.5%	25.0%	-2.5%	-0.081	3.8%	2.5%	1.3%	0.1795
11	28.3%	10.0%	18.3%	0.7086	27.5%	5.0%	22.5%	1.0471	28.8%	12.5%	16.3%	0.5896
12	32.5%	31.7%	0.8%	0.0233	75.0%	65.0%	10.0%	0.2892	11.3%	15.0%	-3.8%	-0.177
13	31.7%	12.5%	19.2%	0.6733	40.0%	17.5%	22.5%	0.6812	27.5%	10.0%	17.5%	0.6838

Table S2. Memory Performance by response type, related to STAR Methods. Memory performance was calculated comparing the rate of remembering scenes (R) with the remember false alarms (RFA) for both emotional (e) and neutral scenes (n). A 2x2 repeated measures ANOVA revealed a significant main effect of memory ($F(1,8)=13.1$, $p=0.0068$) but no interaction ($F(1,8)=0.33$, $p = 0.58$) or effect of stimulus type ($F(1,8)=3.5$, $p = 0.098$, emotional vs neutral). Performance was above chance level for both eR – eRFA (PR eR), $t(8) = 3.72$, $p = 0.0059$) and nR – nRFA (PR nR), $t(8) = 3.00$, $p = 0.0171$. Analyzing d' prime yielded similar results and we found an average d' of 0.496, 0.526 and 0.515 for all, emotional, and neutral images, respectively. We found a trend towards a higher percentage of emotional false alarms (eRFAs: 20.6%) compared to neutral false alarms (nRFAs: 7.6%, $t(8) = 2.02$, $p = 0.0777$), an effect that was significant when analyzing the performance of a larger cohort of patients with and without microelectrodes (Costa et al., 2022).

We excluded 3 patients from all neuronal analyses for abnormal or poor behavioral performance. Patient 2 made most responses around 500 ms (data not shown), coinciding with stimulus offset and only chose Know once. Patients 10 and 12 were excluded for poor memory performance because they had PR and d' values near zero. All behavioral differences were similar in the reduced sample (Table S2). We did not detect any neurons in Patient 11, and therefore, all neuronal analyses were performed in 5 patients (blue rows).

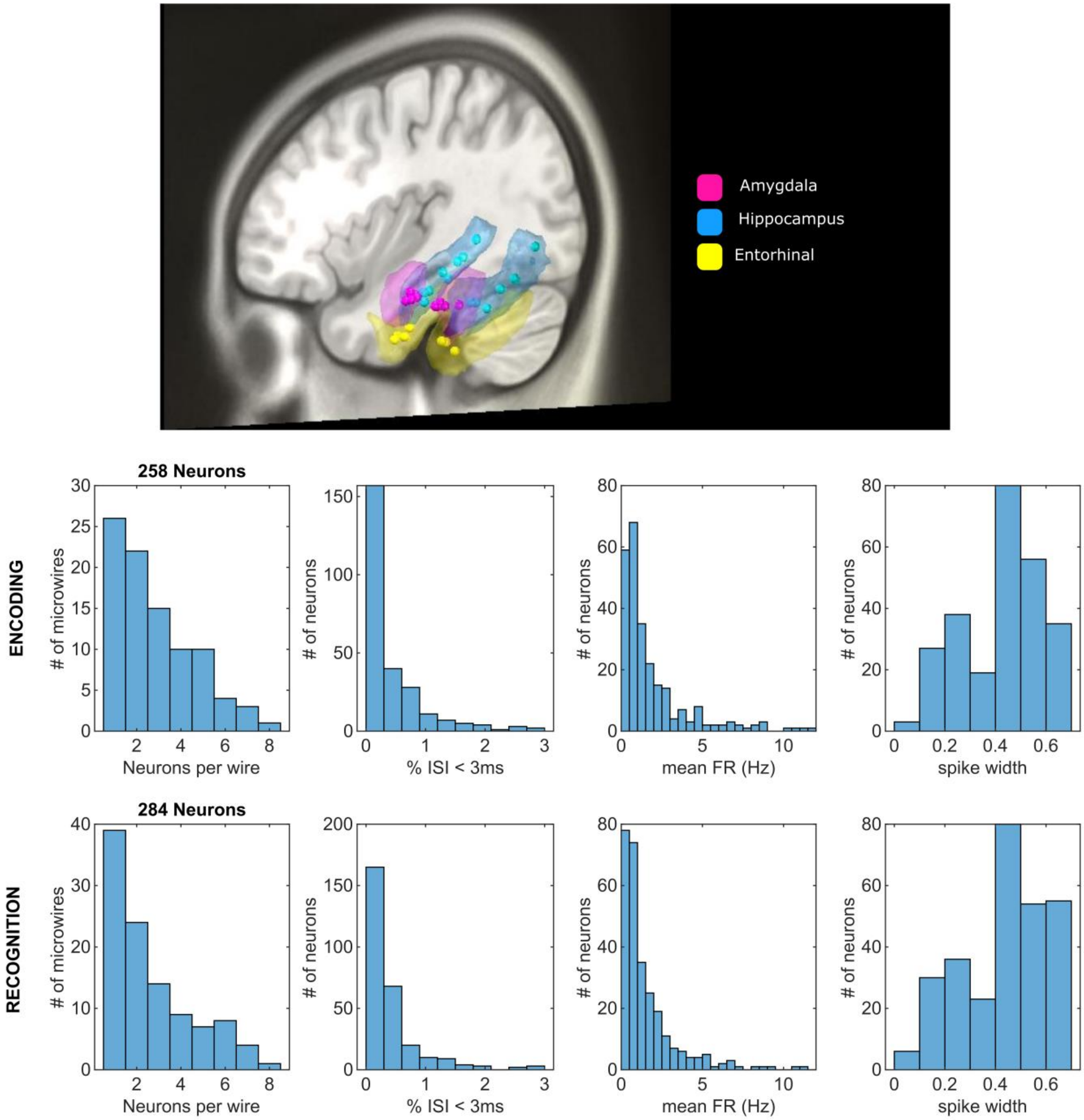

Figure S1. Microwire localization and spike sorting quality metrics, related to STAR Methods. Microwires from patients included in neuronal analyses were located in the hippocampus, amygdala and entorhinal cortex. Only one neuron was detected on most microwires, but few microwires had up to 8 neurons. All neurons analyzed had zero ISIs < 3ms. Two encoding and 4 recognition neurons are not shown in the third column because they had firing rates above 12 Hz but are present in the 3 other columns. Spike width = peak – trough in ms. FR: Firing rate, ISI: Inter-spike Interval.

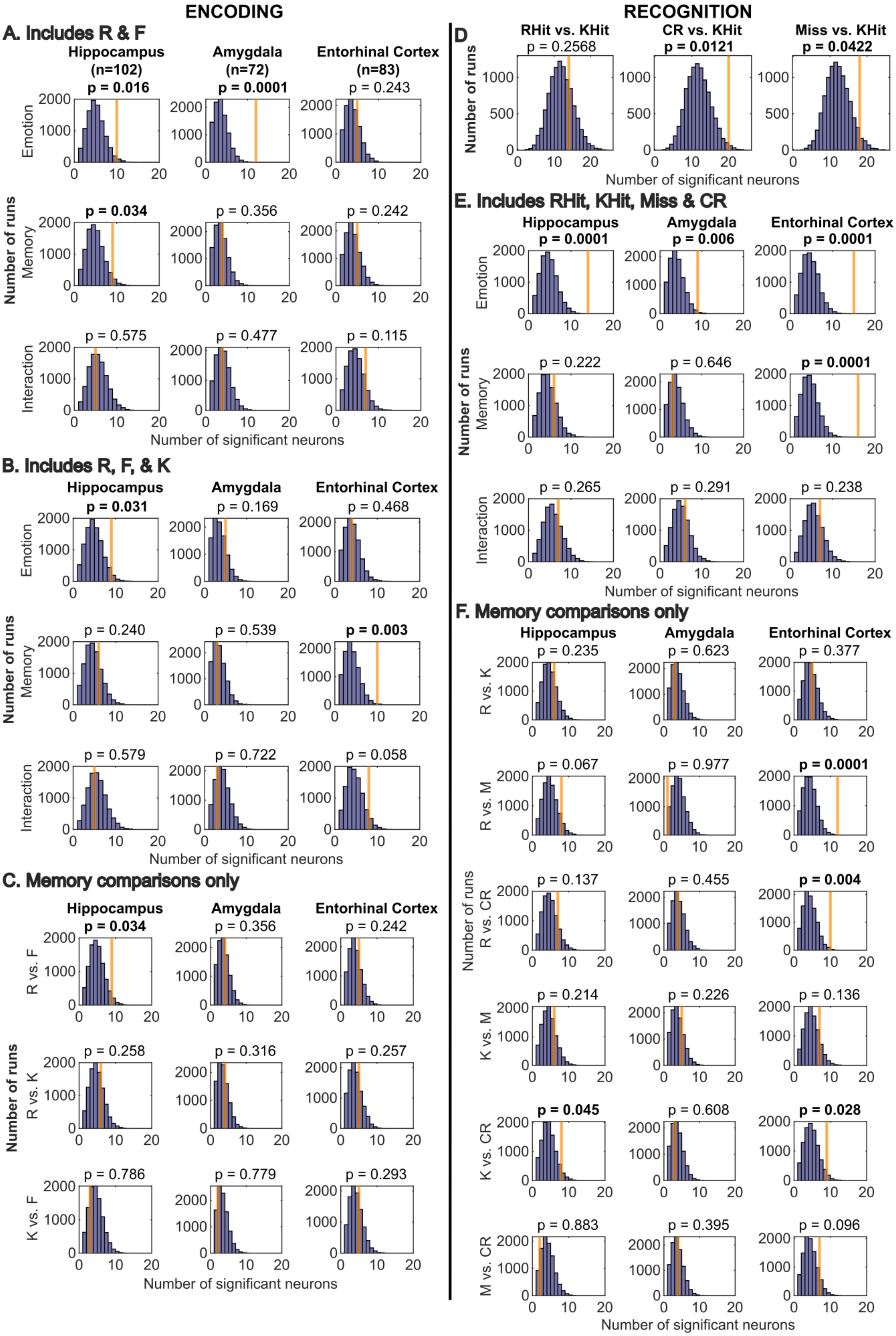
ENCODING
A. Includes R & F
Hippocampus (n=102) p = 0.016
Amygdala (n=72) p = 0.0001
Entorhinal Cortex (n=83) p = 0.243
Emotion
Memory
Interaction
p = 0.034
p = 0.356
p = 0.242
p = 0.575
p = 0.477
p = 0.115
Number of runs
Number of significant neurons
B. Includes R, F, & K
Hippocampus p = 0.031
Amygdala p = 0.169
Entorhinal Cortex p = 0.468
p = 0.240
p = 0.539
p = 0.003
p = 0.579
p = 0.722
p = 0.058
C. Memory comparisons only
Hippocampus p = 0.034
Amygdala p = 0.356
Entorhinal Cortex p = 0.242
R vs. F
R vs. K
K vs. F
p = 0.258
p = 0.316
p = 0.257
p = 0.786
p = 0.779
p = 0.293
RECOGNITION
D
RHit vs. KHit p = 0.2568
CR vs. KHit p = 0.0121
Miss vs. KHit p = 0.0422
E. Includes RHit, KHit, Miss & CR
Hippocampus p = 0.0001
Amygdala p = 0.006
Entorhinal Cortex p = 0.0001
p = 0.222
p = 0.646
p = 0.0001
p = 0.265
p = 0.291
p = 0.238
F. Memory comparisons only
Hippocampus p = 0.235
Amygdala p = 0.623
Entorhinal Cortex p = 0.377
R vs. K
R vs. M
R vs. CR
K vs. M
K vs. CR
M vs. CR
p = 0.067
p = 0.977
p = 0.0001
p = 0.137
p = 0.455
p = 0.004
p = 0.214
p = 0.226
p = 0.136
p = 0.045
p = 0.608
p = 0.028
p = 0.883
p = 0.395
p = 0.096

Figure S2. Bootstrapping statistics for number of neurons by brain region and trial type, related to Figure 2Eand 2F-H. Significant p-values are bolded in all titles. **(A)** Bootstrapping statistics for all subjects comparing Remembered to Forgotten trials. Main effects of emotion were detected in the hippocampus and amygdala. A main effect of memory was detected in the hippocampus and an interaction in the EC. R: Remembered, K: Know, F: Forgotten. **(B)** Bootstrapping statistics including Know (K) trials revealed a main effect of memory in the EC. **(C)** Bootstrapping statistics for the main effect of memory by region showed that the hippocampus distinguished remembered from forgotten trials. **(D)** Bootstrapping statistics for all 3 MTL regions combined comparing KHits to other trial types. P-values are uncorrected but would be nonsignificant after applying Bonferroni correction for 6 comparisons with a significance threshold of $p < 0.0086$. **(E)** Bootstrapping statistics comparing all 4 trial types (RHits, KHits, Misses, Correct Rejections). A main effect of emotion was detected in all brain regions, and a main effect of memory was detected in the EC. **(F)** Bootstrapping statistics for the main effects of memory by region. The hippocampus distinguished RHits from Misses and KHits from CRs. The EC distinguished RHits from Misses, RHits from CRs, KHits from Misses, and KHits from CRs. R: RHits, K: KHits, M: Misses, CR: Correct Rejections.

MTL neurons increased their firing rate in response to visual stimuli during encoding and recognition, but other recent results with an overlapping dataset did not find this [1]. The likely reason for this difference is that here we used Combinato [2] for spike sorting, while WaveClus [3] was used in the previous manuscript [1]. In this dataset, Combinato yielded better isolated clusters and automatically removed simultaneously occurring noise on multiple channels, which drastically improved the quality of isolated neurons.

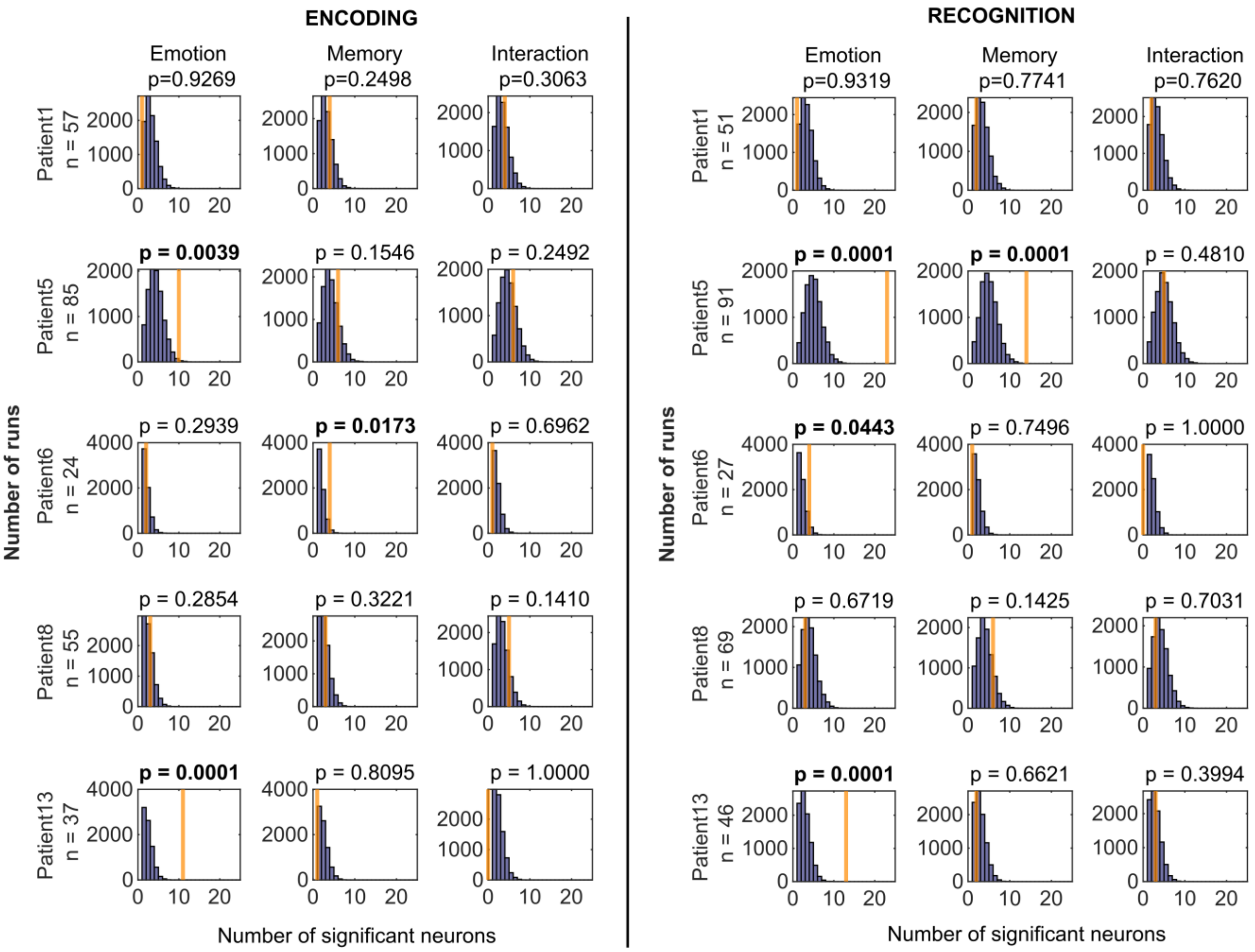

Figure S3. Bootstrapping statistics for number of neurons by subject, related to Figures 2E & 2F-H. Significant p-values are bolded. Bootstrapping analyses revealed 3 out of 4 main effects in the patient with the most neurons, patient 5, further supporting the conclusion that effects would likely be detected with larger samples.

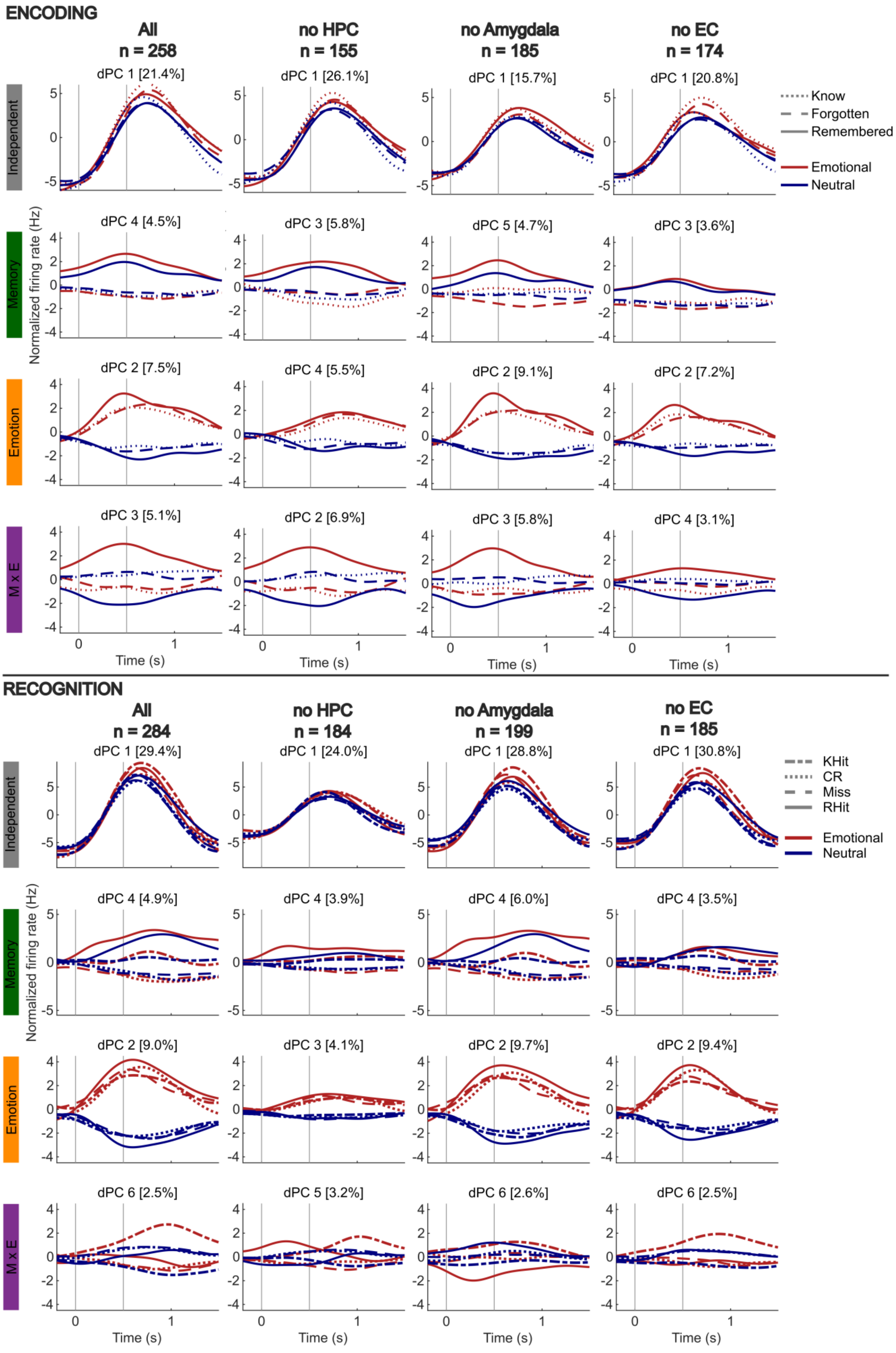

ENCODING
All
n = 258
no HPC
n = 155
no Amygdala
n = 185
no EC
n = 174
dPC 1 [21.4%]
dPC 1 [26.1%]
dPC 1 [15.7%]
dPC 1 [20.8%]
Know
Forgotten
Remembered
Emotional
Neutral
Independent
Memory
Emotion
M x E
Normalized firing rate (Hz)
dPC 4 [4.5%]
dPC 3 [5.8%]
dPC 5 [4.7%]
dPC 3 [3.6%]
dPC 2 [7.5%]
dPC 4 [5.5%]
dPC 2 [9.1%]
dPC 2 [7.2%]
dPC 3 [5.1%]
dPC 2 [6.9%]
dPC 3 [5.8%]
dPC 4 [3.1%]
Time (s)
RECOGNITION
All
n = 284
no HPC
n = 184
no Amygdala
n = 199
no EC
n = 185
dPC 1 [29.4%]
dPC 1 [24.0%]
dPC 1 [28.8%]
dPC 1 [30.8%]
KHit
CR
Miss
RHit
Emotional
Neutral
dPC 4 [4.9%]
dPC 4 [3.9%]
dPC 4 [6.0%]
dPC 4 [3.5%]
dPC 2 [9.0%]
dPC 3 [4.1%]
dPC 2 [9.7%]
dPC 2 [9.4%]
dPC 6 [2.5%]
dPC 5 [3.2%]
dPC 6 [2.6%]
dPC 6 [2.5%]
Time (s)

Figure S4. Leave-One-Out demixed PCA including Know trials, related to Figures 3B & 4B. To qualitatively compare emotional and memory processing in each brain region, we compared dPCA results obtained from the entire dataset to analyzing the dataset without each brain region separately. During both encoding and recognition, the emotion effect was reduced most strongly by removing the hippocampus, the region with the most neurons. The overall absence of dramatic differences supports the idea that the entire MTL is involved in cooperatively performing emotional memory on the single-unit level.

REFERENCES

1. Costa M, Lozano-Soldevilla D, Gil-Nagel A, et al. Aversive memory formation in humans involves an amygdala-hippocampus phase code. *Nat Commun*. 2022;13(1):6403. doi:10.1038/S41467-022-33828-2

2. Niediek J, Boström J, Elger CE, Mormann F. Reliable Analysis of Single-Unit Recordings from the Human Brain under Noisy Conditions: Tracking Neurons over Hours. *PLoS One*. 2016;11(12):e0166598. doi:10.1371/JOURNAL.PONE.0166598

3. Chaure FJ, Rey HG, Quian Quiroga R. A novel and fully automatic spike-sorting implementation with variable number of features. *J Neurophysiol*. 2018;120(4):1859-1871. doi:10.1152/JN.00339.2018/ASSET/IMAGES/LARGE/Z9K0091847690007.JPEG
